# HIV-related stigma in the UK then and now: to what extent are we on track to eliminate stigma? A qualitative investigation

**DOI:** 10.1186/s12889-021-11000-7

**Published:** 2021-05-30

**Authors:** Barbara Hedge, Karrish Devan, Jose Catalan, Anna Cheshire, Damien Ridge

**Affiliations:** 1 University of Westminster, London, UK; 2 South London & Maudsley Trust, London, UK; 3 South Kensington and Chelsea Mental Health Centre, CNWL NHS Trust, London, UK

**Keywords:** HIV, Acquired immunodeficiency syndrome, Qualitative research, Social stigma

## Abstract

**Background:**

The introduction of effective antiretroviral treatment in the late 1990s led to the perception that HIV was a chronic but manageable condition. Nevertheless, stigma remains one of the major hurdles for people living with HIV (PLWH) to accessing healthcare and biomedical preventions. Thus, *Fast Track Cities* has set a target of zero HIV discrimination by 2030 as part of its strategy to end HIV transmission.

**Methods:**

Fifty-three participants from the United Kingdom, including PLWH (*n* = 21, 40%), health and social care workers (*n* = 24, 45%), and charity workers and activists (*n* = 13, 25%), were recruited. Semi-structured interviews investigated stigma and discrimination, focusing on both before and after the widespread use of effective antiretroviral treatment in the late 1990s. Data were analysed using a thematic approach.

**Results:**

Before effective antiretroviral treatment narratives were shaped by two main themes: 1) the media’s role in influencing public opinion and contributing to misunderstandings of HIV transmission; and 2) personal experiences of HIV-related stigma, which for PLWH included incidents of physical violence and aggression, as well as fears of their HIV status being publicised. Contemporary narratives on stigma experiences were organised around four themes: 1) discrimination in healthcare settings; 2) stigma amongst men who have sex with men (MSM); 3) stigma towards African and Afro-Caribbean PLWH; and 4) the limits of change in public HIV-related knowledge and attitudes. Contemporary narratives indicated a reduction in enacted stigma, but continued anticipation of discrimination and self-reported shame, particularly in MSM and African and Afro-Caribbean PLWH.

**Conclusion:**

The nature of stigma against those with HIV has evolved. The intersection of PLWH and minority groups (e.g. MSM and African and Afro-Caribbean persons) may enhance anticipatory and internalised stigma, with some suggestion that this may contribute to reduced engagement in HIV care and prevention services. Our findings indicate the need for further research in this area, as well as proactive interventions with community groups to enhance knowledge of HIV.

**Supplementary Information:**

The online version contains supplementary material available at 10.1186/s12889-021-11000-7.

## Background

Stigma was described by Goffman as a personal quality that is considered to discredit the individual [1]. Stigma has been associated across the ages with new, poorly understood or untreatable conditions like mental illness, leprosy and HIV. Earnshaw and colleagues applied a framework to the classification of stigma experienced by people living with HIV (PLWH) as enacted, anticipated, and internalised [2]. Enacted stigma refers to the actual experience of the negative reactions directed at the individual from societal members. Anticipated stigma signifies the discrimination expected by individuals, while internalised stigma represents the beliefs individuals adopted about themselves. Studies of PLWH suggest that those reporting higher levels of internalised stigma have poorer health [3]. In addition, community stigma, which refers to a person’s perceptions of the severity of stigmatizing attitudes that exist socially, has been found to interact with internalized stigma to produce adverse outcomes for PLWH [4].

Stigma impacts on PLWH, who report feeling inferior in comparison with the rest of society, leading HIV stigma to be considered an additional dimenstion to the AIDS epidemic [5]. Early surveys reported members of the public believing that PLWH deserved their illness and would consider quarantining them [6], and many expressed uneasiness at the thought of close proximity to PLWH [7, 8]. In the UK, one factor that appears to have contributed to discrimination against PLWH during the early days of the HIV epidemic was the high level of fear of a new, fatal disease that was considered generally infectious or contagious [7]. This portrayal was driven by the media accompanied by a popular morality that already discriminated against minority groups, such as men who had sex with men (MSM) and people who injected drugs (PWID) [9, 10]. Research also suggested that the physical signs of some advanced sequelae of HIV (such as visible Kaposi’s sarcoma lesions, severe weight loss, and widespread fungal infections), allowed PLWH to be readily identified, and thus discriminated against [11]..

With adherence to effective combination antiretroviral therapies (ART) introduced in the late 1990s, PLWH can now achieve an undetectable viral load resulting in near-normal life expectancy and, importantly for tackling stigma, persuasive evidence of an inability to transmit the virus [12]. We might, therefore, expect stigma towards PLWH to fade away, and as knowledge about the virus has increased, public support for radical measures, such as quarantine, to decrease. But discrimination against PLWH continues. In the UK, evidence suggests that many still experience stigma and discrimination from a variety of sources ranging from healthcare workers and employers to friends [13]. Despite medical advances, it appears that stigma remains a barrier for many PLWH to accessing care [14]. Increased levels of stigma are associated with a reluctance to get tested or to engage in HIV treatment [15]. HIV-related stigma continues to be associated with reduced adherence to medication and increased psychological symptoms [16–18]. Some PLWH have reported a high level of fear of discrimination from healthcare workers [19, 20]. Stigmatizing attitudes in health workers are frequently related to poor knowledge about HIV [21], and, there is still stigma attached to the mode in which an individual is thought to have acquired HIV e.g. via anal sex [22].

It appears that stigma is frequently heightened by pre-existing inequalities that an individual may be facing, where the intersection with a marginalized-group identity plays a key part in HIV-related stigma [23]. For example, being an MSM or a woman who is also a PLWH can magnify the stigma experienced when accessing healthcare [24], and those with a mental illness may experience increased stigma due to negative attitudes towards their sexuality and gender [23]. Especially high rates of violence towards PLWH have been reported, from intimate partners, among those already stigmatised within society, such as Black, Asian, and Minority Ethnic (BAME) groups [25, 26]. Given all the above evidence, it has been suggested that to reduce an individual’s stigma burden requires identifying and working with the full range of socially devalued characteristics [27, 28].

Individuals associated with PLWH also report what is defined by Goffman as ‘courtesy’ stigma, when the fear of contamination or being discredited extends from PLWH, to their families, friends, and healthcare workers [29, 30].

The *Fast-Track Cities* initiative is an attempt by a global collaboration of cities particularly affected by HIV, together with partners including the United Nations Programme on HIV/AIDS (UNAIDS) and the International association of Providers of AIDS Care (IAPAC), to end the HIV epidemic by 2030. In order to meet its objective, its targets are for 90% of PLWH to know their status, 90% of those diagnosed to receive ART, and of those 90% to sustain viral suppression by 2030; recently a further aim was added: that no one with HIV should experience discrimination (https://www.iapac.org/files/2020/09/Paris-Declaration-3.0-December-2019-1.pdf). However, while there has been much research into the sequelae of stigma on PLWH, especially in the early days of the epidemic, little research has examined narratives of PLWH and their carers regarding perceived changes to HIV-related stigma over time. We need to improve our understanding of the stigma faced by the HIV community in order to establish what is needed to eliminate HIV-related discrimination. Thus, our UK-based qualitative study sought to understand and compare experiences of stigma between the early 1980s and more recent times, through an analysis of narratives of PLWH and those working with them during the last 30 years.

## Methods

### Design

The study was qualitative using one-to-one narrative interviews to collect personal stories around HIV. Qualitative data was selected as the ideal method to gain in-depth data on the topic, and one-to-one interviews allowed us to secure detailed personal narratives about a sensitive topic. Narratives provide people with overarching means to create and transmit meaning in everyday life, and they compel us to listen, as well as to consider the moral dimensions of experience [31].

### Participants and recruitment

Participants were purposely recruited to sample as wide a range of experiences as possible, including the role played in the UK HIV epidemic, the length of time working or living (or both) with HIV, and diverse health specialties. Table 1 describes the demographics, role, and HIV status of the participants. Inclusion criteria were that participants should be 18 years old and over, and able to attend an interview in London or in a location to which the researcher could feasibly travel to given limited resources available (in reality this meant living in the UK mainland), and to have had considerable experience (10 years or more) with HIV. Exclusion criteria were that a participant lived outside the UK or was experiencing severe mental illness.

**Table 1:** Participants’ demographics, roles in epidemic and HIV status (where given)

Demographics	N (%)	N (%)	N (%)
	Male	Female	Total
	36 (68)	17 (32)	53
Ethnicity			
White	35 (97)	13 (76)	48 (91)
African/Afro-Caribbean	1 (3)	2 (12)	3 (6)
South East Asian/Indian	0 (0)	2 (12)	2 (4)
HIV Status			
Positive	19 (53)	2 (12)	21 (40)
Negative or not reported	17 (47)	15 (88)	32 (60)
Role^a^			
Health Professional	14 (39)	10 (59)	24 (45)
Activist	9 (25)	0 (0)	9 (17)
Charity Worker	3 (8)	1 (6)	4 (8)
PLWH	19 (53)	2 (12)	21 (40)
Other^b^	6 (17)	4 (24)	10 (19)
Mode of transmission^c^			
MSM	14 (39)	0 (0)	14 (26)
Heterosexual	1 (3)	2 (12)	3 (6)
People who inject drugs	2 (6)	0 (0)	2 (4)
Haemophilia	2 (6)	0 (0)	2 (4)

^a^Participants may have more than one role

^b^Journalist/Politician/Clergy/Academic

^c^Self-reported as most likely

Participants were initially recruited via the professional and research networks of the authors, and later using snowball sampling whereby existing participants recommended others, to complete our purposive sampling framework. Our final sample comprised PLWH, healthcare professionals, those involved in HIV activism or charity work, clergy, politicians and HIV-related policy makers. A total of 53 participants agreed to be interviewed following an email invitation from the authors.

### Procedure

Interviews were conducted between April 2016 and November 2019 by a consultant clinical and health psychologist (BH, PhD), a consultant psychiatrist (JC, FRCPsych), and a health psychology researcher (AC, PhD). Two interviewers (BH & JC) had long (over 30 years) experience of working within the HIV field, and the latter (AC) had extensive experience in conducting interviews to elicit personal narratives. Interview guides were developed in consultation by the team to encourage participants to tell the story of their life, beginning with living and/or working within the HIV field. Guides were based on approaches to narrative interviewing that suggest encouraging participants to ‘tell their story’ provides one of the richest sources of qualitative data [31]. However, as some participants may find it difficult to narrate, asking about specific topics may also be useful during interviews. Therefore, our interview schedule encouraged story telling with the opening question ‘can you tell me something about life just before HIV?’ and prompts, such as ‘and what happened next?’. In addition, a list of topics was included in the schedule which could be used if the participant did not spontaneously cover a topic of interest to the study. For example, ‘can you tell me about experiences of discrimination?’ This approach was taken for all participants, however, where necessary, some prompts were shaped to match participant’s status (PLWH/health professional/activist); for example ‘thinking more about the present day, what is it like living with HIV now? (PLWH)/what is the care for PlWH like now (health professional)’. Other prompts were unique to participant type, e.g. ‘has stigma associated with HIV prevented you accessing care?’ (PLWH). See Additional File 1 for an example.

All interviews were recorded and transcribed verbatim by a professional transcriber who had signed a confidentiality agreement. Transcripts were checked for accuracy and anonymised by AC, and returned to the participant for checking, with the accepted transcript entered into the database for analysis.

### Analysis

Data were analysed iteratively and inductively [32], using a thematic approach [33], for themes on stigma and discrimination. NVivo 11 software was used to explore and ask questions from the data. Emerging key categories were elaborated, discussed and debated by the authors, to arrive at a robust draft analysis and understanding of the results. All authors were subsequently involved in drafting, re-drafting and iteratively finalising the manuscript.

## Results

### Stigma before effective antiretroviral treatment

Participants with experience of the early days of the UK HIV epidemic reported alarming instances of stigma that were summarised under two main themes: (1) Public perceptions of HIV and the media’s role in developing and maintaining stigma and (2) Experiences of stigma.

### Public perceptions of HIV and the media’s role in developing and maintaining stigma

Participants perceived an inital lack of public and community understanding of HIV. They highlighted misunderstandings of how HIV could be transmitted, associated with fears of catching HIV. Often non-risky acts, such as sharing crockery or even touching a PLWH would lead to fear of transmission. The media were perceived as contributing substantially to the development and maintenance of stigma. The written words and language used by journalists, especially those writing for tabloid newspapers, were believed to be divisive and reinforcing of negative public perceptions about PLWH. Words that de-humanised or degraded PLWH were reported as commonplace in the mainstream media, as noted by a senior HIV doctor:

*Most of the rather sensational reporting on HIV was done not by medical correspondents and it was around contagion, around gay men, about excluding them and herding them up.*

Additionally, the press appear to have divided PLWH into the ‘guilty’ - those whose behaviour was said to have brought HIV infection upon themselves, such as homosexuals and PWIDs, and the ‘innocent’ - those who had been given the virus, such as people with haemophilia or those who acquired HIV as a result of a blood transfusion. An HIV nurse told us about his anger at the media’s reporting:

*Press reports were appalling…today it would be seen as a hate crime. Not just the press, someone senior in the police force said they’re rolling around in a cesspool of their own making…and a member of the royal family said 'it’s the innocent victims I feel sorry for; for the others it’s a self-inflicted wound'.*

Participants highlighted how the media encouraged sensationalism about HIV, with newspapers hunting for celebrities or prominent individuals with HIV who they could expose. Amongst professionals, there was a real fear that journalists would find out their patient’s HIV status and publicise it. This account from an HIV clinical psychologist is characteristic of the kind of running battle with the press:

*Someone in the GP practice had worked out that one of the doctors was positive and had sold the story to a newspaper but [an HIV] charity helped us overnight successfully to get an injunction to stop the story.*

However, participants also reported that one positive consequence from the media interest was increased exposure for individuals who were under-represented in mainstream press (for example MSM), who now appeared in the mass media, explaining clearly and accurately, the facts about living with HIV. Some participants felt that this may have helped to reduce the stigma against PLWH, and under-represented groups, as reported by a psychiatrist:

*In the early ‘80s and ‘90s when so many articulate, presentable, people with HIV were in the media, on TV or the radio, [this] had a huge impact in terms of normalising homosexuality and HIV, making it not such a terrible thing, it’s not, people don’t have horns and look like monsters. That was very influential, and may have played a part in changing attitudes not just to HIV but also to same sex behaviour.*

### Experiences of HIV stigma

Events relating to active discrimination and attacks against PLWH were narrated frequently by participants. Often these were physical acts of violence or aggression, unprompted or seemingly at random, as described by a nurse working in an HIV hospital team:

*People think you exaggerate but I did have patients who had to leave their flats because they had bricks through their windows.*

Along with the physical violence came the violence of ‘othering’ of PLWH by public and social institutions. Here, stigma took the form of a physical separation of PLWH in shared public spaces, such as hospitals. Often this was related to the perception that PLWH were highly contagious. This form of segregation led to participants reporting feeling de-humanised. Sometimes, the behaviours observed in health institutions further added to the experience of discrimination, as described by this HIV nurse and a hospital doctor:

*This young guy had come in...the cubicle door had a little square bit of glass and some of the other patients’ relatives kept peering in, looking at the AIDS patient, and I lost my temper because I was so emotional and I said ‘he’s not an animal from a zoo’.*

*Women who delivered their babies in the maternity hospital…were saying nurses and doctors would come in gowned in space suits, and auxiliaries would open the door, put the food on the floor and then shut the door.*

In addition to the institutional perpetuation of stigma, participants believed that institutions such as prisons, were also responsible for many forced disclosures, with people made to carry around a symbol of having the virus, as a former PWID recalls:

*In prison when we had our (menstrual) periods we had to take bleach everywhere…and if a toilet was used, we had to wash it…Confidentiality right out of the window.*

Partners of people who had died from HIV sometimes experienced rejection by the relatives of the dead person, in particular if the nature of their relationship had not become clear until after the death, as described by an HIV clinical psychologist:

*There had been cases of gay men’s partners being thrown out of their houses because they had no rights when the initial person died, and families taking things back.*

Despite instances of stigma in healthcare, it was via healthcare that many PLWH experienced moments of empathy and human connection. Participants pointed out that such moments occurred rarely in their lives, highlighting the stigma they faced daily. An HIV nurse described this moving example of empathy:

*And I said, 'that food looks nice'. He looked at me and very gingerly said would I like some? I took some and his eyes were full of tears...then I realised he was crying because I had shared his food - he was so used to being treated like a leper.*

Health professionals caring for PLWH also experienced incidences of HIV-related discrimination, as reported by a nurse working with PLWH in the first quote below. Such attitudes appeared to be linked to a lack of understanding and fear of HIV, as a nurse working with PLWH described with the reaction of another nurse in her GP practice in the second quote:

*There was a time when they didn’t want us in the hospital canteen: they wanted us in a separate place and they were saying, should these people be in here?*

*She asked me what I did and I said. She kind of recoiled from me. Almost in horror, and said, aren’t you frightened?*

In some cases, HIV stigma extended not only to the professionals, but to the companies or brands they worked for. Participants reported that there was the implication from healthcare institutions that HIV tainted their public image, as explained by a senior hospital doctor:

*I personally was told to give up my interest in HIV as this was 'ruining' the reputation of the hospital.*

### Contemporary experiences of HIV stigma

Four stigma related themes were developed from the narratives of the participants regarding contemporary experiences of HIV stigma: (1) Stigma in healthcare; (2) Stigma among MSM with HIV; (3) Stigma among Africans and Afro-Caribbeans with HIV; (4) Limited changes to public knowledge and attitudes about HIV.

### Stigma in healthcare

Health care professionals commented on the contribution of clinicians to the continuation of discrimination and stigma. In the words of an activist doctor:

*It is pervasive, and it is something that has to be constantly battled.*

A senior hospital doctor reflected on some of the tensions within hospital teams, describing a reluctance to care for HIV patients often based on a fear of getting HIV themselves. Whilst a senior HIV nurse suggested that it might be lack of familiarity with HIV that drives clinicians to stay away from testing their patients:

*I think testing has got better, and people are more accepting of being tested…it is actually the health professionals that are less willing when it is outside their area (of clinical experience).*

In the opinion of several participants, stigma affects the numbers coming forward for HIV testing, as explained by a senior nurse:

*There is still so much stigma…without it more people would come forward for testing, we would diagnose more of the undiagnosed…it surprises me that the…Stigma Index report for 2015 is as shocking as the one from 2009. It surprises me that even in our city [London] which is supposed to be tolerant and trendy, we are seeing more and more issues around stigma and discrimination.*

The call for the continuation of the safety provided by the HIV specialist clinics is made clear in the views of this activist PLWH:

*Of all the illnesses you could think of, HIV has so much stigma attached to it, that the importance of making the clinics places where you feel safe, and wanted, and valued, is particularly important, if you don’t want to drive people away. And if you drive people away, you don’t get adherence to PrEP and you have the epidemic increasing. So, it’s completely counterproductive and it isn’t cost-effective.*

### Stigma among MSM with HIV

Participants reported that one of the main issues currently facing PLWH is society’s changing attitudes towards the MSM community. Participants acknowledged that there is increasing acceptance of MSM, but were less clear whether this is associated with a reduction of stigma towards PLWH. What does appear to be persistent, is the underlying belief that HIV is a punishment for being MSM. Participants reported they often perceived the intersection of stigma associated with being both gay and a PLWH:

*And even if the mums and dads are great, the fear is ‘Oh my god, but you’ll become HIV positive’ is still one of the most common sentences gay men hear when they come out.*

MSM participants perceived a lack of understanding of their needs from services they required, and expressed concerns that to engage with them, they needed to conceal aspects of themselves. An older PLWH talked about his concerns for the future:

*It’s about growing old as a gay man. There is a fear of having to go back in the closet, who’s going to look after me? The care home is not going to be gay friendly, let alone HIV friendly.*

The concept and expression of safer sex has changed in recent times, potentially impacting on stigma. The availability of biomedical approaches to reduce the risk of acquiring HIV, such as pre-exposure prophylaxis (PrEP) and post-exposure prophylaxis (PEP), added options to the safe expression of sexuality. In addition, thanks to effective ART, PLWH can achieve an undetectable viral load making them unable to transmit HIV even when not using a condom. However, it is still unclear how this new context within which safer sex can be expressed, will influence HIV stigma*.* One participant reported experiencing discrimination from the gay community when he still wanted to use condoms in casual sexual encounters.

*I’d say, 'can you use a condom', and he’d say, 'why?' Because I don’t want to get AIDS, and people would refuse to have sex with you unless it was bareback.*

In the experience of one HIV doctor, some people claimed to be using PrEP in order to avoid disclosing being a PLWH (and associated stigma):

*Even gay men who are undetectable and know they are non-infectious sexually, they don’t tell people that they are undetectable. They just say they are on PrEP because they feel less stigmatised telling others, they are on PrEP rather than telling them they are positive and undetectable.*

Interestingly, while the availability of effective prevention interventions, such PrEP, was feared to be a potential source of public disapproval and stigma, according to an activist PLWH, such negative reactions proved difficult to provoke:

*We live in a country where there is a huge fear of immigrants, there is a lot of racism…but we are not ferociously anti-gay in the way other countries seem to be, so I think we were lucky that the tabloid newspapers didn’t make a huge big thing about PrEP, actually, I think they did try and it didn’t work.*

### Stigma among Africans and Afro-Caribbeans with HIV

Participants highlighted that those with intersecting minority identities, including being a PLWH and African or Afro-Caribbean, were under pressure to conceal multiple stigmatising characteristics, whether this be their HIV status, sexuality, ethnicity or a combination of these. Participants reported fears of stigma from public institutions, especially when accessing services, as one African PLWH summarised:

*I think it’s the narrative in politics about who is sponging off the system. I know people who were born in this country, but because they are Black their HIV status can be an issue…whether it’s accessing benefit or housing, or going to the job centre…They fear that people who are not Black could stigmatise them because of the immigration issue, and then if they know, the HIV.*

For others, fears of stigma often resulted in isolation and avoidance of some social groups. With HIV being considered a taboo issue in some communities, some participants feared that knowledge of their HIV status could be leaked to their local community. For those who were also MSM, anticipated stigma increased. The concerns impacted on access to medical care, as one HIV doctor explained:

*I think a lot of African PLWH will still not disclose their HIV status to family and friends because of stigma. They won’t go to clinics where people work that they know, they won’t register at GP practices where they know people from their community or their country.*

### Limited changes to public knowledge and attitudes about HIV

Participants discussed how knowledge and attitudes towards HIV had improved since the early days, but perhaps not as much as expected, given that some, particularly those in already marginalised groups, continued to experience social ostracization or even acts of aggression. A PWID described his recent experience:

*I thought [attitudes] had changed, but this guy beat me up 3 weeks ago and he said 'you’re riddled with AIDS, you and your junkie pals, you’re riddled with AIDS'.*

For both the community and PLWH, some were concerned that a lack of education in young people was allowing such attitudes to continue:

*The response back in the 80s to the distribution of leaflets around the country with tombstones, shocking, it set the tone for the stigma that carried on for the next 30 years…It set up the frame for a blame culture…it’s an 'us and them culture that has had an insidious effect on things that should be normal and neutral.*

*A social work student was asked ‘Are you not worried about your kid?’ You know, somehow this student is going to catch HIV from being a social worker and then giving it to her kid!*

## Discussion

*Fast Track Cities* aims to end HIV transmission and related stigma by 2030. To shed light on this aim, our study sought a detailed understanding of the current stigma experienced by PLWH in the UK, and how this contrasted with previous experiences, by examining the narratives of PLWH and those working with them. Our study revealed that the success of treatment and improved prognosis for those with HIV infection in the UK, sometimes regarded as the ‘normalization’ of HIV, have been accompanied by changes in the way stigma manifests and is perceived by PLWH and those involved in their care. Participants largely reported less enacted stigma than that experienced in the early days, with a reduction in the intensity of acts of discrimination, but for PLWH from marginalised groups, such as MSM and BAME, internalised and community stigma remain particularly problematic.

PLWH reported feeling uncomfortable in healthcare settings, particularly in GP practices where patients may be known to the staff and the local community. They were concerned about possible breaches of confidentiality as well as discrimination. While this is in line with previous research from the UK [19], US [6], and from South Africa [34], our study has shown that many PLWH still experience enacted or anticipated stigma in the form of institutional or social dishonour. Although our study found that ‘courtesy’ stigma (i.e. the stigma towards healthcare workers and others involved in the care of PLWH) that was prominent in earlier days, is much less evident, care services were perceived by some as playing a role in maintaining many aspects of stigma. Consistent with reports from the US [35], and Iran [21], anticipated or enacted stigma perpetrated by healthcare staff in the UK was seen as reducing the likelihood of PLWH disclosing their HIV status, with the true numbers of PLWH being less visible in healthcare. At the same time, a reduction in deaths attributed to HIV has resulted in HIV falling down the public agenda, and may have contributed to the disbanding of support networks for PLWH.

While enacted HIV sigma prevalent in the early days is now considered to be less frequent and intense, participants noted a continuation of internalised stigma. Our study supports previous research [14–16, 19], that links internalised HIV stigma to compromised healthcare. Here, research has shown that internalised HIV-related stigma is associated with a reluctance to engage with HIV testing services, disengagement with medical services, and at-risk individuals disengaging from HIV prevention services. As previous studies have suggested, internalised HIV stigma can be linked to memories of the early fears and probable prognosis for those diagnosed with HIV. Participants, especially those involved in HIV activism, reported that strong visual images of the early public health campaigns had left a stubborn legacy that continues to contribute to HIV related stigma. Participants hypothesised that young people still form their views on HIV from historical rather than current realities. Participants suggested that the trauma and messaging surrounding the early HIV epidemic remain key drivers of HIV stigma. This concurs with arguments that the initial beliefs an individual gathers about HIV can contribute to later internalised stigma [36].

Our study suggests that while enacted stigma against PLWH has probably reduced, residual stigma is present nowadays, especially for marginalised groups in society who also happen to have HIV, supporting the view that stigma is often compounded when PLWH have other characteristics that bring them into 'disrepute' [24]. Our study was able to identify some communities where PLWH still face enacted stigma. Specifically, the British Black African and Afro-Caribbean participants in this study continued to report experiencing stigma from their local communities, linked both to their sexuality and to their HIV status, in addition to that towards their racialised community. Despite the described increased acceptance within UK society of MSM, sexuality remained a key theme for participants. They saw that, particularly for older people, the potential of becoming infected with HIV remained a negative aspect of being identified as MSM. Stigma directed at – and internalised by – MSM was regarded as still relevant today. This is, for example, evident in the use of PrEP, where stigmatisation by health professionals, and internalised shame on the part of MSM, can lead to a toxic mix, whereby MSM may avoid the use of an effective preventative treatment, and professionals may be reluctant to offer the medication free of judgement [37].

Although our data refer to the stigma still experienced by PLWH in the UK, there are reports from many countries including South Africa, Iran and Ethiopia [38–40], that indicate its negative effects are widespread. Our research indicates that reducing HIV related stigma, although necessary, is a complex task, involving both HIV-related factors (e.g. eliminating outdated perceptions of prognosis of an HIV diagnosis, and challenging memories of images from initial public information campaigning), as well as more systemic factors, such as the ‘othering’ of minorities, especially at a time of economic and political turmoil. Additionally, our data suggest that continuing to monitor levels of HIV stigma needs to be part of the process of evaluating and campaigning for its (hopeful) decline. The impact on the general public of earlier publicity campaigns could be mitigated by instigating positive campaigns and information that highlight the success of antiretroviral medications for PLWH, and the importance of access to treatment. There is good evidence for the efficacy of information-based interventions, skills building approaches, and the use of opinion leaders [41]. Future research should investigate the effect such interventions have on stigma, help-seeking and treatment adherence.

Tackling stigma in healthcare settings would require improvements in education and training. There is evidence that the involvement of stigmatised individuals in the training of healthcare workers may reduce stigma [42]. Future research needs to seek a detailed understanding of health professionals’ knowledge in this area, in order to identify where knowledge is lacking. Given that many PLWH in the UK are now managed in primary care, this would appear to be a good area for initial investigations [43]. Clearly, efforts to reduce HIV stigma need to be part of a broader systemic effort towards tackling discrimination against minorities, based on ethnicity, gender, sexuality or health status [23, 28]. Finally, our study found a reduction in courtesy stigma. It would be useful for future research to explore whether this is associated with an increasing acceptance of PLWH or their increased invisibility.

### Limitations

This study does have a number of limitations. As the researchers facilitated recruitment of participants through their personal and professional contacts, this may have restricted the range of opinions reported. However, two of the researchers (AC and DR) were not part of the UK history of HIV, and so they were able to suggest participants, interrogate the data and conduct analysis as relative outsiders. As these HIV stigma narratives were collected as part of a wider study on HIV, a subsequent study focusing solely on HIV stigma experiences may yield more in-depth data. Finally, the large majority of participants were White, therefore the views of Black, Asian, and other ethnicities may not be fully represented by our research.

## Conclusions

Our study provides some data that can support the *Fast Track Cities* 2030 plan to end discrimination by highlighting where efforts could best be focused. PLWH from already discriminated communities, such as MSM or BAME, still report stigma. These enduring and residual pockets of stigma will need to be addressed if we are to end the epidemic. Internalised stigma, with a lineage to memories of the early epidemic and public health campaigning, remains to be eliminated as it is associated with PLWH (and those at risk of becoming infected with HIV) being reluctant to engage with HIV care and associated services. There are some positive signs. For example, courtesy and enacted stigma are reported to have diminished. However, participants were unclear whether this is related to the increasing acceptance and/or the increased invisibility of PLWH. This appears to be a potential fruitful avenue for future research.

## Supplementary Information

**Additional file 1.** Interview guide: example of our study’s interview schedule, questions and topics.

## Data Availability

The datasets during and/or analysed during the current study are available from the corresponding author on reasonable request.

## Abbreviations

ART: Antiretroviral therapy
BAME: Black, Asian, and Minority Ethnic
IAPAC: International Association of Providers of AIDS Care
HIV: Human Immunodeficiency Virus
PWID: People who inject drugs
MSM: Men who have sex with men
PEP: Post-exposure prophylaxis
PLWH: People living with HIV
PrEP: Pre-exposure prophylaxis
UNAIDS: United Nations Programme on HIV/AIDS
UK: United Kingdom
