# Supplementary material for: HIV-related stigma in the UK then and now: to what extent are we on track to eliminate stigma? A qualitative investigation
Source: BMC Public Health. 2021 May 30;21:1022. doi: 10.1186/s12889-021-11000-7 (PMC8166014; doi:10.1186/s12889-021-11000-7)
Supplement: Supplementary file 1 — Additional file 1. Interview guide: example of our study’s interview schedule, questions and topics. [file 12889_2021_11000_MOESM1_ESM.docx]

**CHANGES IN THE CARE & SUPPORT FOR PWHIV**

**INTERVIEW SCHEDULE – PATIENTS**

**INTRODUCTION**

- Thank participant for taking part.
- Introduce self and the project.
- Then provide information about the interview:
  - I am interested in your stories about the care and support for PWHIV/AIDS in the UK, from when you were first affected by HIV until the present time, focusing on what communities, NHS, social services, and voluntary organizations, provided (whether they be good, lacking, novel or just terrible!).
  - I would like to know about your experience and/or that of people close to you. There are no right or wrong answers, we just want to build a picture of what it has been like from your point of view.
  - Say how long it will take, check how long they have, remind them they can take break if they want, finish the interview on another day.
  - I would like to record the interview, and unless you are happy for your name to be identified, it will be treated in complete confidence.
- Any questions?
- Check participant is happy to start

**INTERVIEW GUIDE**

**Part A - Getting the narrative**

*Opening question*

I’m here today to get your ‘story’, particularly about your experiences of the care and support available to people with HIV from community, NHS, social services, and voluntary organizations over the years.

Can you tell me something about life just before HIV?

*Topics to cover*

Can you tell me about the time that you found out about HIV?

Prompts (all bulleted questions are prompts to encourage the participant to talk further on the topic, to be used if needed)

- What made you decide to get tested?
- What was involved in having the test?
- How did it feel when you received your diagnosis?

What happened after testing for you?

- If you did become ill at or after testing, can you talk about that?
- If didn’t become ill: did you have any concerns about developing symptoms/becoming ill (e.g. with dementia)
- What was your experience of having HIV at this time?
- What was it like dealing with illness?
- What was the prospect of dying like? (prospect of own or that of friends)
- What was your experience of your friends/family?
- What was your experience of community, social and voluntary services (including support for mental health)?

What was your experience of the NHS at this time?

- (If relevant) What treatment options were available to you?
- Ask about both in-patient and out-patient experiences
- (if relevant) what was it like on the HIV wards? (including visits to friends/partner)
- What was your experience of receiving psychological support?

When did you first hear about combination therapy (1995/96),

- What was happening for you around then? (they may not have ever gone on them if they didn’t need them).
- What changes did the introduction of combination therapy make for you?
- Did you think about having treatment or about refusing it?

What happened for you after receiving effective antiviral treatments?

- What effect did it have on your working life?
- What was the effect on people with HIV that you knew?

What happened for you in the years after combination antivirals were introduced?

Thinking more about the present day, what is it like living with HIV now?

- Compared with beginning?
- What are the key challenges in your life living with HIV?
- What health and social care services are available for PWHIV now?
- Who provides your care?
- How do you cope living with HIV?
- Experiences using social and voluntary services?
- How do you feel now about your own mortality?

What is NHS care like for you these days?

- What has changed (better/worse)?

**Part B - Additional questions**

What were relationships like between patients and health professionals? Has this changed over time?

What do you feel could be improved in relation to current service provision?

Have the attitudes of society influenced the care you have accessed for HIV over the year

- Has stigma associated with HIV prevented you accessing care?

Has anything positive come out of the care you received for HIV?

- Anything learnt
- Altered perspectives on life
- Effects on relationships

Is there anything else you want to say, but you have not had a chance to say it?

Notes

Most important is we want to know about medical, community, voluntary and social care, and how this changed over time

If possible, check with HWHIV which time periods they are referring too

Explore political involvement and activism/changes as it comes up
